# Drug toxicity in the proximal tubule: new models, methods and mechanisms

**DOI:** 10.1007/s00467-021-05121-9

**Published:** 2021-05-28

**Authors:** Andrew M. Hall, Francesco Trepiccione, Robert J. Unwin

**Affiliations:** 1 Institute of Anatomy, University of Zurich, Winterthurerstrasse 190, 8057 Zurich, Switzerland; 2 Department of Nephrology, University Hospital Zurich, Zurich, Switzerland; 3 Department of Translational Medical Science, University of Campania ‘Luigi Vanvitelli’, Naples, Italy; 4 Biogem Research Institute, Ariano Irpino, Italy; 5 Department of Renal Medicine, University College London, London, UK

**Keywords:** Proximal tubule, Drug toxicity, Fanconi syndrome

## Abstract

The proximal tubule (PT) reabsorbs most of the glomerular filtrate and plays an important role in the uptake, metabolism and excretion of xenobiotics. Some therapeutic drugs are harmful to the PT, and resulting nephrotoxicity is thought to be responsible for approximately 1 in 6 of cases of children hospitalized with acute kidney injury (AKI). Clinically, PT dysfunction leads to urinary wasting of important solutes normally reabsorbed by this nephron segment, leading to systemic complications such as bone demineralization and a clinical scenario known as the renal Fanconi syndrome (RFS). While PT defects can be diagnosed using a combination of blood and urine markers, including urinary excretion of low molecular weight proteins (LMWP), standardized definitions of what constitutes clinically significant toxicity are lacking, and identifying which patients will go on to develop progressive loss of kidney function remains a major challenge. In addition, much of our understanding of cellular mechanisms of drug toxicity is still limited, partly due to the constraints of available cell and animal models. However, advances in new and more sophisticated in vitro models of the PT, along with the application of high-content analytical methods that can provide readouts more relevant to the clinical manifestations of nephrotoxicity, are beginning to extend our knowledge. Such technical progress should help in discovering new biomarkers that can better detect nephrotoxicity earlier and predict its long-term consequences, and herald a new era of more personalized medicine.

## Introduction

**Fig. 1:**
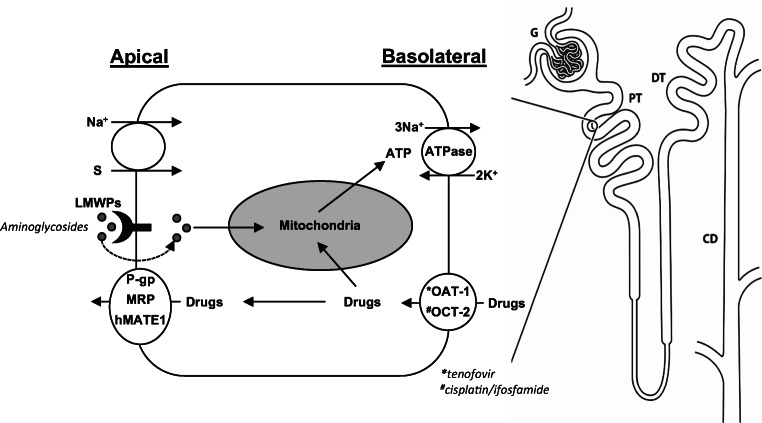
Drug transport in the proximal tubule. The reabsorption of filtered solutes (S) across the apical membrane of the proximal tubule (PT) is coupled to that of sodium (Na^+^), utilizing intracellular gradients generated by the basolateral Na^+^/K^+^-ATPase. Drugs are taken up from the blood via basolateral organic anion and cation transporters (OATs and OCTs, respectively) and excreted into the urine via apically expressed ABC transporters, such as P-glycoprotein (P-gp), multidrug resistance proteins (MRPs) and human multidrug and toxin compound extrusion-1 (hMATE1). Receptor-mediated endocytosis provides an apical entry route for filtered drugs such as aminoglycosides. Some drugs passing through PT cells are toxic to mitochondria, and can disrupt the supply of ATP to the basolateral Na^+^/K^+^-ATPase, resulting in a breakdown in transport and wasting of solutes in the urine (renal Fanconi syndrome). *DT* distal tubule, *CD* collecting duct, *G* Glomerulus

The kidney proximal tubule (PT) has two major functions. First, to reabsorb the majority of the glomerular filtrate and some specific solutes, including small low molecular weight proteins (LMWP), amino acids, phosphate, glucose and bicarbonate, for which transport processes in the distal nephron are either not present or are of limited capacity. The complete maturation of the full transport capabilities of the PT occurs post-weaning and up to the post-neonatal phase [1]. Second, along with the liver, the PT is a major route by which many non-filtered xenobiotics are metabolized and excreted. To execute the latter function, PT cells express a number of drug transporters and enzymes for both Phase I (oxidation by cytochrome P450s) and Phase II (conjugation to glutathione) metabolism of drugs [2]. Drug transporters expressed in the PT include basolateral organic anion (OAT-1) and cation (OCT-2) transporters that mediate the uptake from blood of potential PT nephrotoxins such as the anti-retroviral tenofovir and the cytotoxics cisplatin and ifosfamide, respectively, as well as apical efflux transporters such as P-glycoprotein transporter (P-gp), multidrug resistance protein (MRP), and human multidrug and toxin compound extrusion-1 (hMATE1), which can determine drug secretion and intracellular accumulation (Fig. 1) [3, 4].

The expression of drug transporters and metabolizing enzymes is generally greater in the more distal parts of the PT (S2 and S3), and this is mirrored by a higher density of peroxisomes in these segments, which play an important role in drug metabolism [5]. Conversely, apical receptor-mediated endocytosis of filtered proteins via the multi-ligand receptors megalin and cubilin is more prominent in the first (S1) part of the PT [6], and this represents another route of entry for drugs like aminoglycosides, which are polycationic and readily attracted to the anionic phospholipid apical membrane that facilitates their interaction with the megalin–cubilin receptor complex [7].

Perhaps unsurprisingly, due to the high number of compounds passing through or accumulating in the PT, it is a common site of drug-induced kidney damage [8–10]. Clinical presentation of patients with drug toxicity in the PT depends on the severity of damage and can range from mild asymptomatic urinary losses of solutes to rapid, life-threatening loss of all excretory function (acute kidney injury—AKI). Studies have suggested that nephrotoxicity is responsible for approximately 16% of cases of children hospitalized with AKI, and up to a quarter of cases of AKI in adults [11]. However, estimates for the prevalence of proximal tubulopathy caused by individual drugs vary widely. For example, kidney dysfunction has been reported in 10–80% of children receiving cisplatin and 1–30% treated with ifosfamide [12]. Studies of kidney toxicity in children exposed to the anti-viral drug tenofovir have also yielded a range of results [13], with one longitudinal study reporting that 4% had to stop therapy due to proximal tubulopathy [14], and more than 20% of adults taking the same drug have detectable PT defects in cross-sectional studies [15].

Heterogeneity in findings among studies is probably explained by a number of variables, including the study population, degree of exposure, drug interactions, duration of follow-up and pre-existing kidney function, as well as pharmacogenetic differences that may alter drug metabolism and/or excretion. A lack of standardized definitions of what constitutes clinically significant tubular toxicity is another problem, although efforts are ongoing as part of the pharmaceutical industry and drug regulatory authorities (FDA and EMEA) Predictive Safety Testing Consortium (PSTC) to try to address this, and approval has already been gained for some early markers of renal tubular injury such as Kidney Injury Molecule-1 (KIM-1) and β_2_-microglobulin [16].

Although it has been recognized for decades that drugs can induce functional defects in the PT, several challenges remain. First, there is a clear need for new and better approaches to predict PT toxicity at an earlier stage in drug development and to reduce its risk [17]. Second, in most cases, the exact cellular mechanisms underlying drug PT toxicity are still not fully elucidated, which makes devising strategies to predict or prevent damage difficult. Third, it remains unclear whether patients with mild-to-moderate PT defects (e.g. an isolated increase in glycosuria or urinary excretion of LMWP) will go on to develop progressive loss of kidney function and declining GFR. That said, a recent publication reporting kidney function from a lifetime cohort study of children treated for childhood cancer found that only around 2% had moderate-to-severe kidney impairment (stages 3–5 chronic kidney disease (CKD)) by the time they were young adults, mainly as a result of historical exposure to platinum-based cytotoxics and/or kidney irradiation [18].

In this article, we will summarize how drug toxicity in the PT can present clinically and how it may be diagnosed and monitored. We will discuss how more sophisticated in vitro models, high-content analytical techniques and novel urinary biomarkers are making it possible for both academic and industry-based researchers to begin to make inroads into some of the existing challenges.

## Clinical presentation of drug toxicity in the proximal tubule

Drug-induced PT dysfunction typically leads to impairment of PT transport, resulting in urinary wasting of certain solutes. Clinical presentation depends on the nature and severity of the insult [7, 8, 10]. At the mildest end of the spectrum patients are asymptomatic, and toxicity only manifests as a modest increase in urinary concentration of one or two solutes (e.g. LMWP), without overt changes in blood parameters. As the magnitude of transport disruption increases, more marked wasting of more solutes occurs, resulting in systemic deficiencies and complications (e.g. hypophosphatemia and bone demineralization), a scenario known as the renal Fanconi syndrome (RFS) [19]. Finally, more severe insults can cause structural damage and/or cell death in the PT, leading to decreases in GFR (possibly due to cell shedding and luminal obstruction) and AKI^1^ [7].

PT dysfunction can be diagnosed using a combination of blood and urine tests. Patients often display hypophosphatemia and metabolic acidosis. Simultaneous measurement of urine phosphate concentration allows calculation of the fractional excretion to confirm that decreases in blood concentration are due to increased renal losses [20]. Serum bicarbonate concentration is usually only modestly decreased, and urinary excretion may be undetectable, if acid excretion in distal tubular segments remains intact; a bicarbonate infusion would be necessary to confirm increased clearance. Serum creatinine concentration can be raised, since it is normally secreted by the PT [21], but it is mainly a marker of glomerular filtration and has limited sensitivity to detect drug-induced PT damage.

Measurement of LMWP in the urine, such as retinol-binding protein (RBP4) and β_2_-microglobulin (see earlier), is frequently used to monitor patients, since increases in excretion (‘tubular proteinuria’) can usually be detected early in PT toxicity [15, 22]. In addition, the magnitude of excretion provides a quantitative assessment of the severity of PT injury [23, 24]. However, the long-term prognostic significance of isolated tubular proteinuria has been called into question by several clinical studies (see later—Patients with isolated tubular proteinuria). Urinary albumin may also be increased in patients with PT dysfunction, since it is partially reabsorbed in the PT. However, unlike LMWP, it is not so freely filtered, and its appearance in the urine in large amounts reflects mainly a defect in glomerular permeability [24]. Therefore, dipstick testing is not a sensitive marker of PT damage and will only show positivity for tubular proteinuria when losses are high.

Amino acids can be measured in urine; although this may be helpful in diagnosing inherited forms of proximal tubulopathy, it is not routinely performed when screening for drug toxicity. Dipstick testing for non-diabetic glycosuria is a quick and easy screen, but this lacks sensitivity due to the high reabsorptive capacity for glucose in the PT and is not usually positive until damage is severe [15].

Other than loss of kidney function per se, a major clinical consequence of PT dysfunction is a decrease in bone density (rickets/osteomalacia) due to phosphate depletion, which can also be aggravated by decreased activation of vitamin D in the PT. Patients may be asymptomatic, and bone health should be considered in any patient with RFS. In severe cases, bone pain may occur, and it was the presenting symptom in several early reports of severe PT dysfunction due to the anti-viral tenofovir [25]. The PT also has an important role in secreting organic solutes that are not freely filtered at the glomerulus because of protein binding. Many of these are waste products of metabolism, and some have been labeled as ‘uremic toxins’ with potentially harmful cardiovascular effects [4], but whether they accumulate in the blood of patients with drug-induced PT dysfunction requires further study. However, while independent estimates of tubular secretory function (using endogenous metabolites such as hippurate) broadly correlate with GFR, which is used to determine dosing for many renally excreted drugs, there is a case to be made for beginning to incorporate measures of tubular secretory capacity in dose adjustment algorithms for some drugs, especially in CKD [26].

Although routinely performed, imaging studies such as kidney ultrasound play a limited role in the investigation of patients with drug-induced tubulopathy. Kidney biopsy is occasionally indicated: for example, if there is significant doubt about the etiology of a sudden and rapid decline in kidney function. This rarely yields critical mechanistic insights, beyond non-specific findings such as tubular flattening and accumulation of luminal debris, but occasionally reveals acute tubulo-interstitial nephritis as an idiosyncratic drug reaction. However, electron microscopy can reveal evidence of mitochondrial abnormalities with drugs such as tenofovir [25, 27].

### Some clinical considerations in pediatric patients

This is most pertinent for children and adolescents receiving chemotherapy for childhood cancers. The most commonly used nephrotoxic drugs causing tubular injury are, as already mentioned, cisplatin and ifosfamide, followed by carboplatin and nitrosureas [12], although the latter drug class is more likely to cause glomerular injury. Renal side effects may develop after single drug administration or in combinations, or as a cumulative effect of single or multiple drug repeat exposures. Other rarely used drugs such as EGF receptor inhibitors, BRAF inhibitors and the newer checkpoint inhibitors seem to cause more widespread tubular toxicity, hypomagnesemia being a particular feature of EGF receptor inhibitors [28]. Moreover, the associated usage of NSAIDs as painkillers can contribute to the development of PT damage [29]. In neonatal rats treated with ibuprofen or indomethacin to recapitulate the indication in pre-term infants to close a patent ductus arteriosus, these drugs were shown to induce severe PT vacuolization, as well as glomerular damage [30].

Unlike glomerular disease, which can usually be detected by routine blood and urine testing, assessment of PT function requires more elaborate blood and matched urine collections to assess renal clearance and secretory capacity, which is more challenging and time consuming. However, there is lack of standardization for testing and values for normal ranges, although this should improve as more tubular biomarkers are identified and validated.

## Cellular mechanisms of drug toxicity in the proximal tubule

There are several reasons why PT cells are generally vulnerable to toxins [9]. First, the kidney receives around 25% of the cardiac output, and thus a high delivery of blood-borne substances. Second, PT cells actively take up drugs via two separate routes: from the blood via basolateral transporters and from the filtrate via apical endocytosis. Third, while metabolism of drugs typically reduces their toxicity, in certain cases, it can produce noxious metabolites, as in the case of ifosfamide [31]. Finally, many drugs are harmful to mitochondria, and PT cells are densely packed with these organelles. Moreover, they are almost entirely dependent on aerobic metabolism to generate the ATP that is required to energize solute transport [32]. However, beyond these well-established potential mechanisms, the exact cellular processes by which drugs cause PT toxicity remain largely unknown, due principally to a lack of appropriate models and methods for investigation.

In broad terms, drugs affecting the PT can be divided into two classes: those in which toxicity can be expected and is dose related (Type A), and those in which it is unexpected and idiosyncratic (Type B). The first group consists of drugs that are intended to be cytotoxic and can damage any cell that takes them up: examples include chemotherapeutics like cisplatin and ifosfamide [33, 34]. Since cytotoxicity is an intrinsic property of these compounds, attempts to mitigate kidney damage are largely focused on reducing PT exposure (e.g. with careful dosing matched to body weight and GFR) or uptake (e.g. with agents that can block drug transporters, such as cimetidine). Downstream effects of mitochondrial toxicity, including reactive oxide species (ROS) generation, may also be targeted to limit cellular damage [35].

Unlike chemotherapy agents, many other drugs are not known to be cytotoxic but may nevertheless display evidence of nephrotoxicity either during development or during post-marketing surveillance. Indeed, nephrotoxicity still accounts for about 10% of safety failures in drug development [36]. Examples of such drugs include anti-virals (tenofovir), anti-bacterials (gentamicin, vancomycin), anti-convulsants (valproate) and iron chelators (deferasirox) [8, 10]. Typically, these drugs have adverse effects on PT cell function (i.e. solute transport), rather than cell viability, but usually reverse on stopping the drug. This suggests that they interact with important metabolic or signaling pathways within PT cells, probably due to unexpected off-target effects. A deeper understanding of what underlies these toxicities should improve drug risk-benefit.

To better elucidate the adverse effects of drugs on PT cell function, analytical methods are required that provide detailed readouts of processes such as transport, metabolism and signaling, and not only cell viability. Examples of this approach include large-scale metabolite profiling using mass spectrometry and nuclear magnetic resonance (NMR) [37], gene expression and proteomic screens [38], oxygen consumption rate (OCR) measurements [39] and live cell imaging [40, 41]. The suitability of each will depend on the drug in question, and they can, of course, be combined to build up a more complete picture. This is important because some drugs may affect more than one cellular process. An example of a multi-modal approach is provided by a recent study that identified multiple pathways altered by cisplatin in human-derived PT cells, including the Nrf2-mediated oxidative stress response, various mitochondrial processes, and AMPK, mTOR and p53 signaling [42]. Furthermore, deployment of tissue-embedded microsensors for oxygen and various metabolites in human kidney spheroids has revealed that glucose uptake is critical to the development of lipotoxicity induced by cyclosporine and cisplatin [43].

### Deferasirox—an example of off-target effects on PT function

Live cell imaging is a particularly attractive method to investigate metabolic drug effects since dynamic changes in both the structure and function of organelles can be followed in real time. One of the authors (AMH) used this approach to identify an off-target effect of the iron chelator deferasirox on the inner mitochondrial membrane (IMM) that may explain its toxicity in humans [44]. Clinical studies and case reports suggest that deferasirox causes PT dysfunction [45–47], which is unlikely to be due to iron depletion per se, since this is not reported with other chelators.

Using confocal imaging in PT-derived cells, it was found that deferasirox, but not other chelators, induced dramatic swelling of mitochondria without de-energizing them [44]. Moreover, it increased OCR but decreased cellular ATP content. Taken together, these findings are consistent with a partial uncoupling effect (i.e. increasing the leak of protons across the IMM, which decreases the efficiency of ATP production), but without fully permeabilizing the IMM. Deferasirox is highly lipophilic (which explains its good oral bioavailability) and a weak acid at physiological pH, and previous studies have shown that other compounds with these chemical properties can also uncouple the respiratory chain by effectively ‘carrying’ protons across the IMM [39]. Mitochondrial swelling was also observed in damaged PTs in mice treated chronically with deferasirox, suggesting that the mechanism also occurs in vivo [44]. Thus, the example of deferasirox demonstrates how off-target effects of drugs can unintentionally cause a functional toxicity and that live cell imaging can play a useful role in uncovering underlying mechanisms.

## New in vitro proximal tubule models to screen for drug toxicity

In the previous example of deferasirox, a well-established PT cell line was sufficient to identify a mechanism of functional toxicity. However, elucidating pathogenic pathways for other drugs will probably require more sophisticated in vitro models that more closely resemble the situation in vivo. For example, PT cells in their native environment perform far more solute transport and endocytosis than in vitro and contain complex drug metabolizing machinery. In addition, they are considerably less glycolytic but display a high level of activity in other metabolic pathways, such as gluconeogenesis, ammoniagenesis, the pentose phosphate pathway and various redox reactions [48], some or all of which might be lost when cells are in culture or immortalized.

The need to recreate this level of complexity, combined with a parallel drive to decrease dependence on animal experiments (for ethical, cost and reliability reasons), has led to intensive efforts in both academia and industry to generate more representative in vitro models of the PT. Recent in-depth articles have reviewed this rapidly expanding field in detail [49, 50], but the main areas of active research include more differentiated human-derived cell lines [51, 52], reprogrammed stem cells [53], kidney organoids [54, 55] and microfluidic devices (so-called kidney-on-a-chip) [56, 57]. Each approach has advantages and disadvantages [49], for example organoids can partially mimic the three-dimensional environment of the normal kidney, allowing the study of cross-talk between PT cells and surrounding interstitial cells in response to injury. However, they are not yet suited to standardized and high-throughput screening necessary for drug development. Luminal flow and apical shear stress have recently been identified as key determinants of PT cell differentiation [58, 59], and the possibility of recapitulating these and also to directly address apical to basolateral solute transport are advantages of the rapid progress in microfluidic devices. Multiple organs can also be represented on a single chip (e.g. liver and kidney, and even intestine) to reconstruct the complexity of more integrated drug metabolism in living animals [60]. Although promising, these methods are still in early development and not sufficiently ‘high-throughput’ but are likely to be scalable over the next few years.

Improved understanding of the transcription factors that drive renal epithelial cell differentiation has recently led to groundbreaking studies in which tubular cells were generated in vitro by directly reprogramming mouse or human fibroblasts [53]. So far, it has not been possible to specifically produce cells in this way that have a ‘pure’ PT phenotype; however, the future potential of this approach for personalized medicine is obvious. Finally, a possible compromise between mammalian and in vitro models can be found in zebrafish larvae. These have a functioning PT in the pronephros, which partially represents the mammalian equivalent. Crucially, zebrafish are amenable to high-throughput studies, including live imaging, which can be used to screen for drug-induced effects on PT function [61].

## Monitoring and management of patients with drug-induced proximal tubular dysfunction

Management of nephrotoxicity can be conceptualized and summarized by the 6Rs: Risk (patient and drug), Recognition, Response (prompt withdrawal), Renal support, Rehabilitation (short- and long-term monitoring) and Research (documentation and wider surveillance) [62]. Patients taking drugs with the potential to cause PT toxicity can be monitored using appropriate markers of PT function as described earlier, such as tubular proteinuria and fractional excretion of phosphate. The frequency of testing should be tailored to the individual patient and drug. Moreover, measurement of drug levels is also important for potent nephrotoxins with narrow therapeutic windows, such as aminoglycosides and calcineurin inhibitors. General risk factors for toxicity include high dosage, older age (in adults), low body weight, pre-existing kidney impairment and interactions with other prescribed drugs [63]. However, it remains almost impossible to accurately predict the chances of toxicity arising in a given patient. This has stimulated a concerted effort to try to understand genetic factors that may explain individual variability in response to drugs.

### Pharmacogenomics

Since the transporters and enzymes responsible for drug handling in the PT are well known, it has been proposed that screening for polymorphisms in the relevant genes could assist in predicting toxicity. However, progress so far with implementing pharmacogenomics into routine clinical practice has been mixed. On the positive side, genetic screening for polymorphisms in cytochrome P450 enzymes responsible for metabolizing the immunosuppressant tacrolimus can guide appropriate dosing in transplant patients [64]. However, although a number of studies have identified associations between genetic variants in drug transporters and the risk of toxicity due to tenofovir [65], these lack sufficient predictive power to adjust doses in individuals, probably because other pharmacokinetic factors determine drug levels in vivo, not least interactions with other drugs excreted via the same pathways.

### Some considerations in pediatric patients

Predicting the likelihood of toxicity and minimizing risk is particularly challenging for pediatric patients, because of a general lack of drug pharmacokinetic studies in children and reliance on extrapolating from adult studies. In addition to considerations of age, body weight and baseline kidney function (GFR), the ontogeny of renal drug elimination also needs to be better appreciated. For example, the activity of many tubular transport mechanisms is low in neonates but subsequently increases rapidly during growth, although at differing rates [66]. Moreover, changes in expression of enzymes important for drug metabolism, such as cytochrome P450s, may underlie the age-dependent risk of toxicity from drugs like ifosfamide [31]. No known treatments favor PT recovery from drug-induced damage, although N-acetylcysteine has been used widely in clinical practice, and there is some evidence for its benefit as an antioxidant [67], but the data in children are limited [68].

### When to stop therapy?

In any given patient with evidence of PT toxicity, the decision to continue or stop therapy will depend on the severity of the defect, the importance of the indication for treatment and the availability of alternative drugs. In cases where the indication is strong (e.g. a life-threatening illness) and other options are limited, this can be a difficult balance to strike, and decisions should be made in consultation with the patient, their family and treating physician. A compromise that is sometimes effective is to simply reduce the dose of drug. PT function usually improves over time on withdrawal of the offending agent, but this can take months and recovery is not always complete [25]. As already mentioned, studies suggest that survivors of childhood cancer have an approximately ninefold increase in risk of developing kidney failure compared to siblings [12].

### Patients with isolated tubular proteinuria

It is not uncommon for patients taking PT toxic drugs to develop mild-to-moderate tubular proteinuria in the absence of other detectable biochemical defects or a decrease in GFR. While it is well established in nephrology that proteinuria of glomerular origin in CKD is associated with an accelerated decline in kidney function, whether tubular proteinuria has similar significance is less clear [69]. In principle, increased concentrations of potentially bioactive peptides in the lumen may induce adverse signaling events further downstream along the renal tubule [70]. However, recent studies performed in patients with tubular proteinuria caused by tenofovir appear to show that this is not associated with any subsequent decline in kidney function [71, 72]. Moreover, some genetic disorders of the PT have been described in the last few years that cause tubular proteinuria, but without any associated loss of GFR [73, 74].

Taken together, these findings suggest that the presence of isolated tubular proteinuria per se may not be sufficient to justify stopping therapy, although it does indicate a degree of tubular dysfunction. However, larger studies are needed with other drugs and of longer duration before tubular proteinuria can be considered to be unharmful. Even if tubular proteinuria is not damaging in itself, loss of these small proteins might have other systemic consequences. For example, decreased reabsorption of vitamin D binding protein may lead to lower blood levels of activated vitamin and affect bone mineralization [75]. The belief that tubular proteinuria may not be deleterious, combined with an appreciation that apical endocytosis is an important entry route for toxins into the PT [69], has led to the suggestion that inhibiting the megalin–cubilin pathway with small molecule inhibitors may be a means of reducing some drug toxicities, for example aminoglycosides (see earlier) and some anti-cancer drugs [7, 76, 77].

### New urinary biomarkers of proximal tubular toxicity

The uncertain predictive usefulness of tubular proteinuria, combined with the insensitivity of measures like estimated GFR and albuminuria, supports the need for newer urinary biomarkers of drug-induced PT damage [78, 79]. Ideally, these would be sensitive enough to detect toxicity at an early stage and, crucially, should be able to discriminate between changes in PT cell biology that may be considered ‘functional’ from those that are harmful and likely to cause progressive kidney injury [80], and provide a more accurate assessment of the true incidence of PT toxicity.

So far, no perfect biomarker exists, although KIM-1 looks promising (see earlier) but is currently more qualitative than quantitative. This transmembrane protein is rapidly upregulated in stressed PT cells following injury and is easily detectable in urine [81]. Recent studies in rats have shown that urine KIM-1 can predict PT injury, confirmed by histopathology, and before a rise in serum creatinine concentration [82]. Many other urinary biomarkers of kidney injury have been explored in the setting of AKI, mainly post-cardiac surgery, and two, TIMP2 and IGFBP7, have emerged and become established recently as early AKI detection tools, including for nephrotoxicity [83]. Both have been approved in combination as a commercially available test (NephroCheck) for AKI risk, but it is unlikely to be specific for PT stress alone [84].

The predictive role of some biomarkers of tubular injury has also been validated in the pediatric population [85], and these are being used in AKI recognition and prevention programs [86]. Urinary biomarkers like NGAL are quick to use and easy to test, with the advantage that they do not subject children to repeated blood sampling; however, they are not yet in routine use but are promising tools for risk stratification in AKI and DIKD [87].

## Conclusion

Drug toxicity is a major cause of disease in the PT in humans, but in most cases, the underlying cellular mechanisms are not known. Thanks to better in vitro models and the application of high-content analytical techniques, we are at last beginning to understand how and why drugs cause functional defects in this crucial nephron segment. However, there is still a long way to go before animals can be reliably replaced for assessing nephrotoxicity and until this can be more easily identified, characterized and predicted in early-stage drug development. Moreover, we lack standardized methods to quantify the severity of PT dysfunction in patients, which is still a problem for clinical studies. We urgently need prognostic markers that can reliably predict long-term effects on kidney health.

Finally, recent findings suggest that the existence of isolated tubular proteinuria may not be a reason in itself for stopping drugs, but patients should certainly be monitored carefully for any signs of worsening kidney function or for systemic complications, including loss of bone density. In the future, integration of pharmacogenomics into clinical practice may predict better the drug response in an individual patient, while newer urinary biomarkers such as KIM-1 might help to distinguish non-progressive changes in PT transport function from more harmful stress responses. Together, these advances could provide a more personalized assessment of the risk of continuing drug therapy.

## Footnotes

1 KDIGO guidelines define 3 phases of kidney injury, AKI (< 7-day duration), AKD (7–90 days) and CKD (> 90 days); drug-induced kidney injury (DIKD) uses a similar classification, although often categorized as acute, sub-acute and chronic, respectively.
